# Blending Self-Assembled Monolayers for Enhanced Band Alignment and Improved Morphology in p-i-n Perovskite Photodetectors

**DOI:** 10.1021/acsami.4c06447

**Published:** 2024-06-19

**Authors:** Edoardo Angela, Davide Nodari, Francesco Furlan, Julianna Panidi, Martyn A. McLachlan, Nicola Gasparini

**Affiliations:** † Department of Materials, Molecular Science Research Hub, Imperial College, London W12 0BZ, U.K.; ‡ Department of Chemistry and Centre for Processable Electronics, Molecular Science Research Hub, Imperial College, London W12 0BZ, U.K.

**Keywords:** perovskite photodetectors, low dark current, self-assembled monolayer, band alignment, specific detectivity

## Abstract

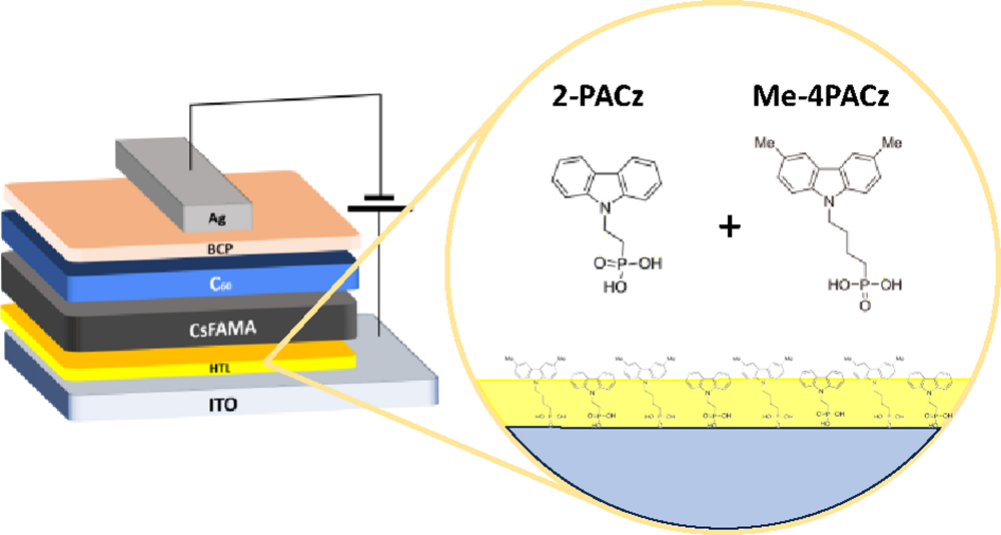

Perovskite photodetectors, devices that convert light to electricity, require good extraction and low noise levels to maximize the signal-to-noise ratio. Self-assembling monolayers (SAMs) have been shown to be effective hole transport materials thanks to their atomic layer thickness, transparency, and energetic alignment with the valence band of the perovskite. While efforts are being made to reduce noise levels via the active layer, little has been done to reduce noise via SAM interfacial engineering. Herein, we report hybrid perovskite photodetectors with high detectivity by blending two different SAMs (2-PACz and Me-4PACz). We find that with a 1:1 2-PACz:Me-4PACz ratio (by weight), the devices achieved a low noise of 1 × 10^–13^ A Hz^–1/2^, a high responsivity of 0.41 A W^–1^ at 710 nm, and a specific detectivity of 6.4 × 10^11^ Jones at 710 nm at −0.5 V, outperforming its two counterparts. In addition to the improved noise levels in these devices, impedance spectroscopy revealed that higher recombination lifetimes of 0.85 μs were achieved for the 1:1 2-PACz:Me-4PACz-based photodetectors, confirming their low defect density.

## Introduction

Photodetectors (PDs) are widely employed in a diverse array of technology platforms including chemical analysis,^1^ medicine,^2−4^ environmental monitoring,^5,6^ defense,^7,8^ imaging,^9,10^ space exploration,^11^ communications,^12−14^ and the Internet of things.^15,16^ Recently, metal halide perovskites have attracted massive interest owing to their high optical absorption coefficients of >10^4^ cm^–1^, long charge carrier lifetimes of >1 μs, diffusion lengths exceeding 1 μm, bandgap energy (*E*_g_) tunability, and solution processability.^17−22^ Such versatility is also reflected in the vast range of optoelectronic devices they embody such as solar cells, LEDs, lasers, and photocatalysts.^23−27^ The specific detectivity (D*) is the main figure of merit in PDs. To achieve high D*, the dark current density (*J*_d_) and consequentially noise current (*i*_n_) should be reduced. The elements causing *J*_d_ are still widely discussed; however, some of its underlying mechanisms such as thermal generation, charge injection, trap-assisted tunneling, and recombination have been identified to largely influence its value.^28−33^ Defects found in the bulk, at the surface, or interfaces of the active layer create defect states within the bandgap, which fuel such mechanisms, thereby increasing *J*_d_.^34^ Defects in the bulk have been attributed to Frenkel defects, dislocations, lattice strain, and migrating ions.^35−37^ Instead, dangling bonds from grain boundaries, anionic vacancies (especially iodine vacancies), and interfaces have been blamed for the surface contribution.^38,39^ Energy level alignment was also shown to dominate the charge injection mechanism: a larger energy difference (Δ*E*) between the anode and perovskite conduction band (CB) suppresses injection and tunneling.^40,41^

Indeed, an abundance of strategies has addressed these issues; chemical and stoichiometric doping were shown to passivate defects in the bulk.^42−45^ Likewise, surface defect passivation has been widely studied by applying buffer layers.^39,46,47^ Charge blocking layers (CBLs) are an important strategy to reduce tunneling and charge injection thanks to the potential barrier posed by Δ*E*. CBLs are now the standard in producing PDs with both organic and inorganic materials being used. However, reactions occurring at the interface and low conductivities limit inorganic and organic HTLs, respectively.^48,49^ Self-assembling monolayers (SAMs) revolutionized the p-i-n perovskite photodiode landscape thanks to negligible parasitic light absorption, perovskite interface passivation, and fast charge transfer.^50,51^ The number of existing SAMs offers a degree of freedom in choosing ones that most suitably align their highest occupied molecular orbital (HOMO) with the perovskite valence band (VB) to extract holes more effectively.^52^ Conversely, the Δ*E* between the SAM HOMO and the perovskite CB has been shown to affect *J*_d_. Larger Δ*E*s can be achieved via SAM choice or by tuning the perovskite *E*_g_ chemically. This enabled an ultralow *J*_d_ of 10^–11^ A cm^2^ and D* values exceeding 10^12^ Jones.^40,53^ Between the pool of SAMs, the [4-(3,6-dimethyl-9*H*-carbazol-9-yl)butyl]phosphonic acid (Me-4PACz) would be a suitable HTL candidate thanks to the alignment of its HOMO level with the perovskite VB. However, triple-cation perovskites present wettability issues due to their nonpolar surface.^54^ Using a SAM blend has been experimented on a NiO layer for photovoltaic applications revealing an increase in surface coverage and a reduction in charge recombination.^52^

Here, we report the incorporation of a blended mixture of two widely studied SAMs, 2-(9*H*-carbazol-9-yl)ethyl]phosphonic acid (2-PACz) and Me-4PACz as an HTL in p-i-n perovskite PDs using triple-cation active layer Cs_0.05_[(FA)_0.83_(MA)_0.17_]_0.95_Pb(I_0.9_Br_0.1_)_3_ (CsFAMA). We adopted a similar approach, which could allow the incorporation of Me-4PACz in a PD as well as allow better overall coverage. This would combine Me-4PACz’s HOMO alignment with fewer interfacial defects. The best-performing PDs were achieved using the blended SAMs, with champion devices featuring an ultralow noise of 1 × 10^–13^ A Hz^–1/2^ and a specific detectivity of 6.4 × 10^11^ Jones at −0.5 V. The blending of the SAMs results in synergistic improvements in perovskite crystallization (and thus morphology) owing to the 2-PACz and a closer band alignment between the perovskite VB and the SAM HOMO driven by the Me-4PACz. This was reflected in higher charge carrier lifetimes of 0.85 μs, which demonstrate a lower defect density. Thus, our result highlights a facile route to enhance performance in PDs by combining SAMs, compared to single molecular species.

## Results and Discussion

**Figure 1:**
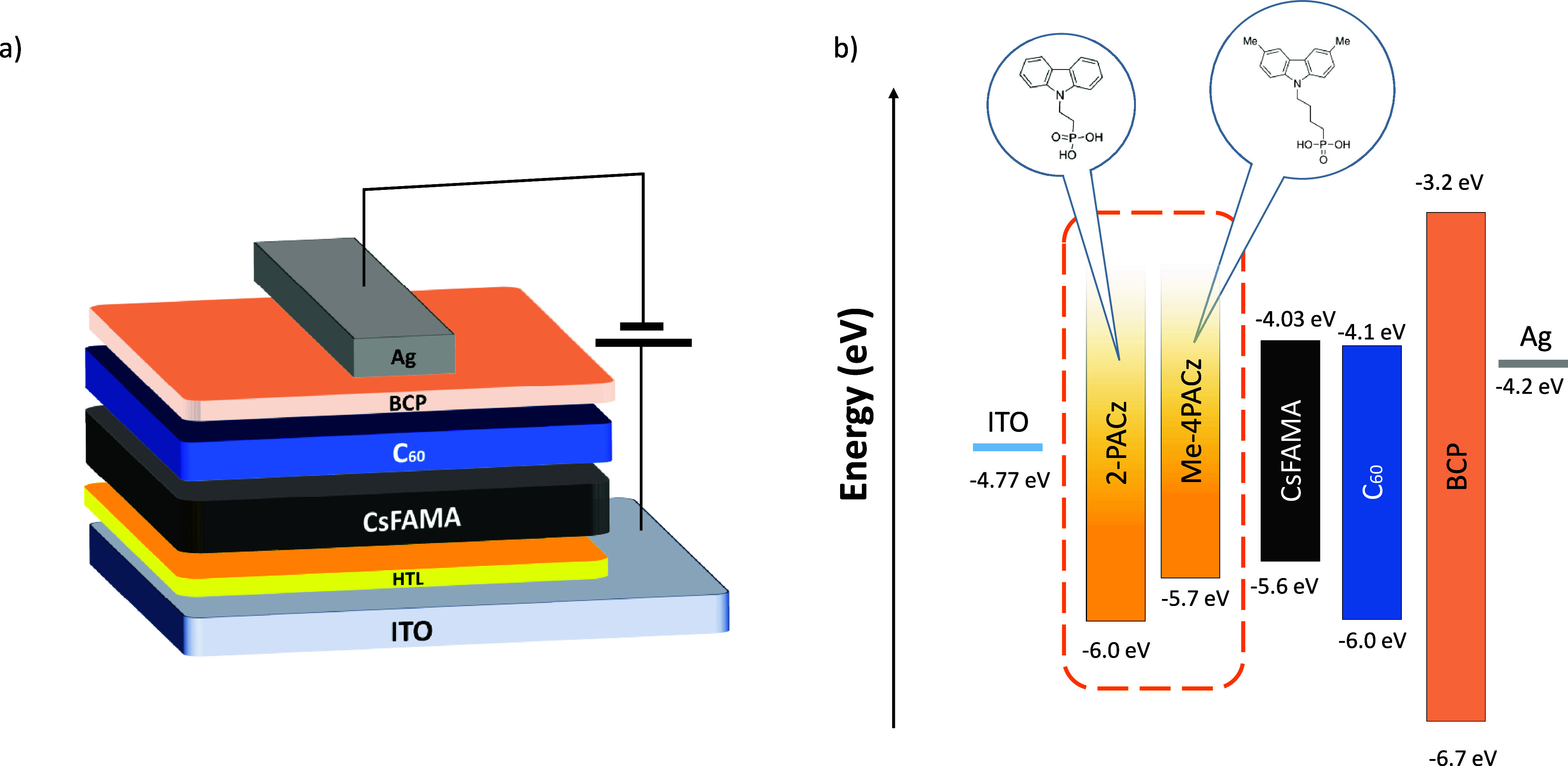
Schematic representation of (a) the p-i-n photodiode architecture and (b) the energy levels and alignment of the various layers, with the chemical structures of 2-PACz and Me-4PACz displayed. The values were obtained via APS and UV–vis.

The devices reported in this work consist of an inverted (p-i-n) PD architecture, as shown in Figure 1a. The perovskite, Cs_0.05_[(FA)_0.83_(MA)_0.17_]_0.95_Pb(I_0.9_Br_0.1_)_3_ (CsFAMA), served as the active layer and C_60_ and bathocuproine (BCP) as the electron transporting and hole blocking layers, respectively. The HTLs used were 2-PACz, Me-4PACz, and their blend at 1:1 by a weight ratio.

The highest occupied molecular orbital (HOMO) levels of the SAMs were found to be −6.0, −5.7, and −5.8 eV for 2-PACz, Me-4PACz, and their blend, respectively, using air photoemission spectroscopy (APS) (Figure S1 and Table S1 in the Supporting Information). Similarly, the valence band edge of CsFAMA was measured by APS at −5.6 eV (Figure S2) and its *E*_g_ of 1.57 eV was determined from UV–vis measurements and its corresponding Tauc plot (Figure S3). From these data, we prepared the flat band energy level diagram shown in Figure 1b, revealing Me-4PACz’s HOMO to be more closely aligned to the perovskite’s VB edge than 2-PACz.

**Figure 2:**
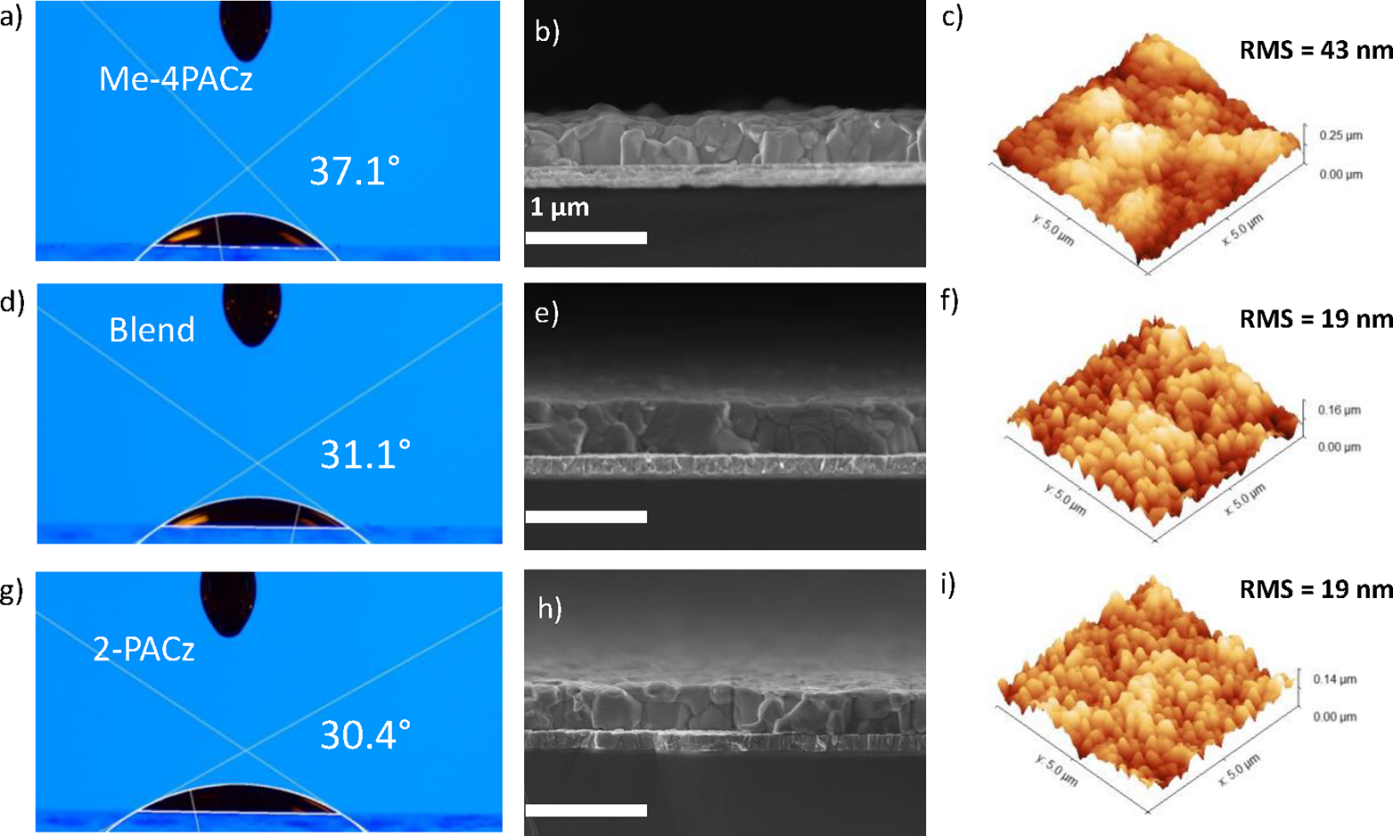
Contact angle images, cross-sectional SEM images, and AFM images for Me-4PACz samples (a–c), blend samples (d–f), and 2-PACz samples (g–i).

In advance of device fabrication, the contact angle of the perovskite precursor solution was measured on the two SAMs and their blend, as depicted in Figure 2a,d,g. The measured contact angles for the Me-4PACz, 2-PACz, and their blend were 37.1, 30.4, and 31.1°, respectively, indicating subtly reduced wettability for the Me-4PACz modified substrate. To quantify perovskite film thickness and examine film morphology, cross-sectional scanning electron microscopy (SEM) images of the active layers deposited on a variety of SAM-modified surfaces are shown in Figure 2b,e,h and top-view images in Figure S4. A relatively consistent perovskite film thickness of 400 nm is observed for all films. However, the perovskite deposited on the Me-4PACz modified substrate shows a nonuniformly flat surface and larger grain size distribution, attributed to the reduced wettability impacting film formation.^55^ This is not observed in the blend case. To evaluate the impact of the SAMs on the perovskite layer, we utilized AFM, as presented in Figure 2c,f,i and Figure S5. The data agree with the SEM cross-sectional images, revealing a rougher surface for the Me-4PACz samples with an RMS value of 43 nm compared to the 19 nm ones exhibited by samples with 2-PACz and the blended SAMs. The morphology was also probed with XRD, showing highly crystalline perovskite films with the characteristic (100) and (110) peaks observed at 14.2 and 20.0°, respectively, for the simple cubic structure^56^ (Figure S6). Thus, we have demonstrated that the blend of SAMs affords the favorable surface energy of 2-PACz to be preserved while incorporating Me-4PACz with its desirable HOMO level. Furthermore, films grown on Me-PACz reveal a rougher surface than its counterparts.

**Figure 3:**
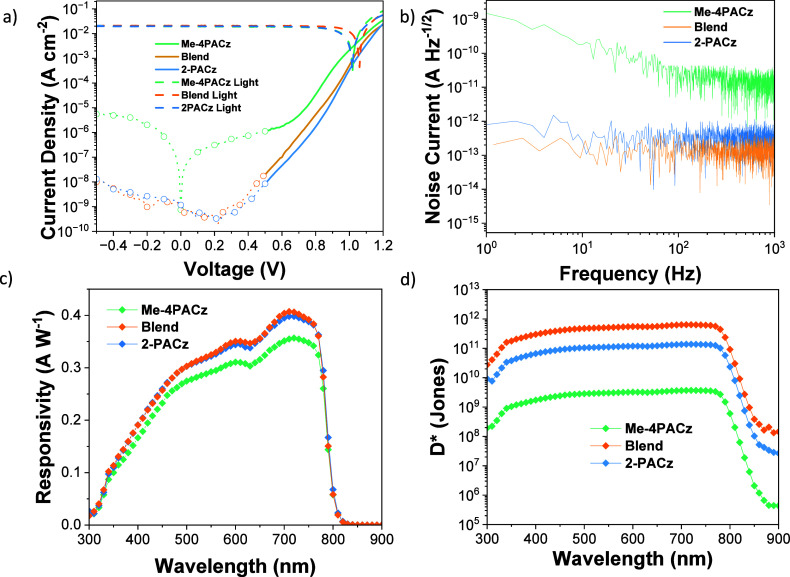
(a) *J*–*V* plots for the different samples in light and dark conditions for the perovskite PDs with the different SAMs. *J*_d_ values were taken at −0.5 V. (b) Power spectral density plot for the different samples over a 1–1000 Hz range. (c, d) Responsivity and specific detectivity plots for the different SAMs.

To ascertain the PD performance, we first determined the dark current density (*J*_d_) by measuring current density–voltage (*J*–*V*) under light and in dark conditions (Figure 3a). The *J*_d_ at −0.5 V for devices prepared using Me-4PACz was the highest measured, at 5.5 × 10^–6^ A cm^–2^, with values of 1.2 × 10^–8^ and 5.4 × 10^–9^ A cm^–2^ obtained for 2-PACz and the SAM blend, respectively (Figure 3a). Statistical variability of the *J*_d_ values can be found in Figure S4, and blend optimization is shown in Figure S7. The *i*_n_ values were not calculated using *J*_d_; instead, they were measured by taking a Fourier transform of *J*_d_ over a time such that a 1–1000 Hz range was obtained. This is exemplified in Figure 3b where devices using Me-4PACz had an *i*_n_ of 2 × 10^–11^ A Hz^–1/2^, whereas the ones with 2-PACz and the blend exhibited lower values of 6 × 10^–13^ and 1 × 10^–13^ A Hz^–1/2^, respectively. Figure S8 shows LDR values of 110.4 and 107.4 dB for 2-PACz and blend, respectively, and 43.4 dB for Me-4PACz. Coherently with the results obtained from the noise, a higher *i*_n_ is usually correlated with lower LDR values.^57^ The samples prepared using Me-4PACz consistently exhibited significantly increased noise compared with 2-PACz and the SAM blend, which suggests that there is a more defect-dense SAM:perovskite interface acting as a center for electrons to be promoted to the perovskite conduction band (CB) via intraband defect states.^58^ The perovskite film morphology can also contribute to the higher *J*_d_, with the distribution of grain size of the perovskite on Me-4PACz being wider than on 2-PACz and the blend. Another factor to consider is the SAM’s coverage of ITO. It has been reported that bulkier SAMs are responsible for a stronger steric effect and thus lower surface coverage.^59^ Mixing the two SAMs together would have given rise to fewer empty areas since 2-PACz would fit where Me-4PACz could not.

The responsivity (*R*) plots for perovskite PDs upon variation of the SAM HTLs are shown in Figure 3c. The spectral profiles show the same features for all layers owing to the use of a common perovskite light absorber, with responsivity tailing off sharply at 810 nm.^60^ The SAM blend and 2-PACz samples show high peak responsivities of 0.41 and 0.40 A W^–1^ at 710 nm, respectively, with Me-4PACz’s achieving only 0.35 A W^–1^. The smaller value found for the Me-4PACz condition indicates that the device had a lower charge extraction than its counterparts.
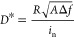
1

PD’s key figure of merit is the specific detectivity (D*). As reported, calculating *i*_n_ from *J*_d_ would overestimate D*,^61^ as shown in Figure S9. D* is described in eq 1, where *A* is the photoactive area and Δ*f* is the bandwidth, considering *R* and *i*_n_ (here, *i*_n_ is taken from the white noise region of Figure 3b). Accordingly, Figure 3d reveals that the blend’s D* of 6.4 × 10^11^ Jones at 710 nm is superior to 2-PACz’s and Me-4PACz’s values of 1.3 × 10^11^ and 3.7 × 10^9^ Jones, respectively.

**Figure 4:**
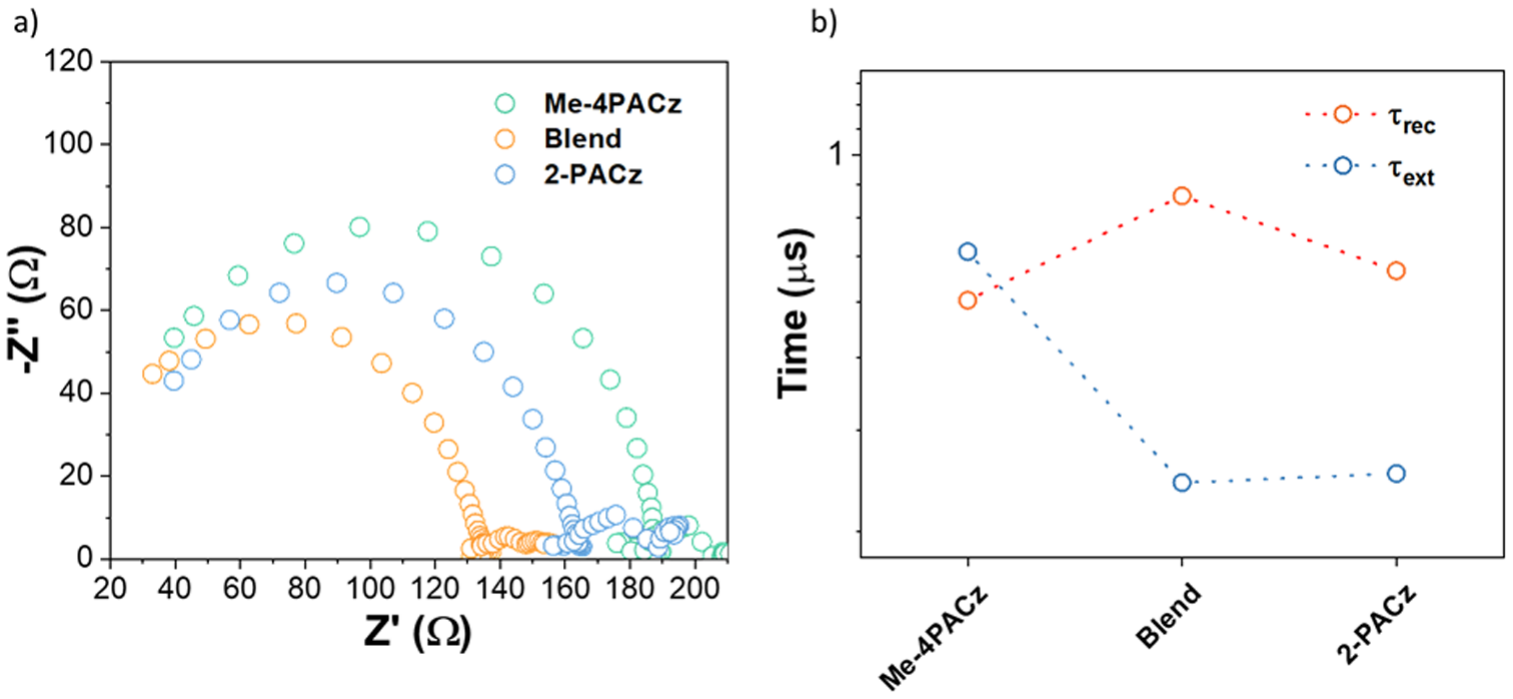
(a) Nyquist plots taken for the devices under green light (530 nm) and a 1.0 V forward bias and (b) recombination and extraction times of the charge carriers. Such values were obtained by fitting the Nyquist plots to an equivalent circuit with the data reported in Table S3. The error was under 5% for the fitted values.

When devices are illuminated and subjected to a voltage equivalent to their open-circuit voltage, the photocurrent and recombination flux cancel each other out. In these conditions, electrochemical impedance spectroscopy (EIS) is a powerful technique to probe for extraction and recombination lifetimes of charge carriers.^62^ Nyquist plots for the devices (Figure 4a) revealed the smallest arc for devices that used the SAM blend and the largest arc for devices with Me-4PACz. An equivalent circuit for the device (Figure S10) was used to fit the Nyquist plots. The series resistance is modeled by the resistor *R*_s_, the recombination resistance *R*_rec_, the extraction resistance *R*_ext_, the geometric capacitance *C*, and the chemical capacitance *C*_μ_ by the constant phase element CPE. Charge carrier lifetimes can be obtained by the product of *R*_rec_ and *C*_μ_ (τ_rec_ = *R*_rec_*C*_μ_) and extraction times by multiplying *R*_ext_ and *C*_μ_ (τ_ext_ = *R*_ext_*C*_μ_).^63−66^ The values are depicted in Table S2.

Figure 4b shows that τ_ext_ is larger than τ_rec_ for the Me-4PACz condition where the process lifetimes are 0.68 and 0.56 μs, respectively. This means that the carriers recombine before they can get extracted, confirming that films with Me4-PACz as an HTL are more defect-dense.^67^ Instead, the opposite is seen under the other two conditions. While the τ_ext_ of ∼0.28 μs is observed in both conditions, devices with blend and 2-PACz showed τ_rec_ values of 0.85 and 0.63 μs, respectively. Therefore, charges are extracted before they recombine as well as having longer lifetimes in devices with the blend HTL. This confirms that blending the two SAMs effectively reduces the defect density in the perovskite active layer. To gain further insight into the reduced defect density, time-resolved PL (TRPL) decay kinetics on perovskite thin films were analyzed. An excitation wavelength of 467 nm and a probing wavelength corresponding to the emission maxima were used. The PL decays are depicted in Figure S11 and Table S3 that collect the average lifetimes (τ_ave_) calculated as per the previous report.^53^ We found that the perovskite film coated on blended SAMs delivered a τ_ave_ of 164 ns, longer than those of 2-PACz (147 ns) and Me-4PACz (134 ns). This confirms the suppressed nonradiative recombination pathways in the blended-based perovskite, indicating reduced defects.

Finally, the reduced defects in blended SAMs delivered faster response speed compared to Me-4PACz-based PD. In particular, we calculated cutoff values of 224, 398, and 355 kHz for Me-4PACz, blend, and 2-PACz, respectively (Figure S12). In line with these values, we extracted the rise and fall plot revealing rise times of 10.4, 2.1, and 1.9 μs for Me-4PACz, blend, and 2-PACz conditions, respectively (Figure S13). Fall times were found to be 11.4, 2.2, and 1.8 μs, respectively.

## Conclusions

In conclusion, we fabricated p-i-n perovskite photodetectors using a blend of SAMs as the HTL. We have demonstrated the beneficial effect of mixing 2-PACz and Me-4PACz in a 1:1 ratio. Samples with the SAM blend revealed an *i*_n_ of 1 × 10^–13^ A Hz^–1/2^ and *R* of 0.41 A W^1–^, resulting in a D* of 6 × 10^11^ Jones. Its 2-PACz and Me-4PACz counterparts achieved 1.3 × 10^11^ and 3.7 × 10^9^ Jones, respectively (Table 1). Overall, the blend outperformed the devices made with pure SAM solutions, with 2-PACz being less performant and Me-4PACz being significantly worse. Our morphological analysis revealed that perovskites that crystallized on Me-4PACz had a larger grain size distribution and an uneven surface, which acted as a major source of defects, which increased charge recombination and noise generation. Blending together 2-Pacz and Me-4PACz allowed one to reproduce a morphology more like that of pure 2-PACz, with a lower defect density. This theory was confirmed by EIS analysis, which revealed τ_ext_ > τ_rec_ for the Me-4PACz condition and τ_rec, blend_ > τ_rec, 2-PACz_. Thus, devices with the SAM blend exhibited the lowest number of defects, via which recombination and noise generation occurred. With this study, we reveal the role of different interfacial SAMs and their synergistic interactions for low-noise and high-detectivity PDs.

**Table 1:** Table Summarizing the Various PD Figures of Merit for the Different Conditions

	**contact angle (°)**	*J*_d_(mA cm^–2^)**@–0.5 V**	**S(A W**^**–1**^**)@710 nm**	***i***_**n**_**(A Hz**^**–1/2**^**)@1000 Hz**	D* (Jones) @710 nm	**τ**_**ext**_**(μs) @530 nm, 1.0 V**	**τ**_**rec**_**(μs) @530 nm, 1.0 V**
Me-4PACz	37.1	5.5 × 10^–3^	0.35	2.0 × 10^–11^	3.7 × 10^9^	0.68	0.56
blend	31.1	1.2× 10^–6^	0.41	1.0 × 10^–13^	6.4 × 10^11^	0.27	0.85
2-PACz	30.4	5.4 × 10^–6^	0.40	6.0 × 10^–13^	1.3 × 10^11^	0.28	0.63

## Methods

Unless otherwise stated, all devices were measured under ambient conditions and chemicals were >99.5% purity and anhydrous. All solvents and materials were purchased from Merck unless stated otherwise.

### Solutions

Cs_0.05_[(FA)_0.83_(MA)_0.17_]_0.95_Pb(I_0.9_Br_0.1_)_3_ solution was prepared by mixing stoichiometric quantities of PbI_2_ (TCI, 99.99%), PbBr_2_ (TCI), and CsI (Sigma-Aldrich, 99.999%) solutions in the stoichiometric ratio, dissolved in a 4:1 mixture of DMF:DMSO (both Sigma-Aldrich), giving a final concentration of 1.2 mol dm^–3^. These solutions were then used to dissolve FAI and MAI (Greatcell Solar Materials, 99.999%).

SAM solutions were prepared by preparing 30 mM solutions of Me-4PACz (TCI) and 2-PACz (TCI) in ethanol; blends were prepared by mixing these solutions in a 1:1 ratio.

### PD Fabrication

ITO-coated glass substrates were cleaned with deionized water in an ultrasonically heated bath for 15 min. The same procedure was repeated with acetone and isopropanol. The substrates were then dried with a N_2_ gun and cleaned by UV ozone for 15 min. The HTLs were spin-coated on the substrates at 3000 rpm (with an acceleration of 1500 rpm) for 30 s and then dried at 100 °C for 10 min. Onto these substrates, the perovskite was subsequently spin-coated at 1000 rpm (with an acceleration of 1500 rpm) for 10 s and then at 5000 rpm (with an acceleration of 1500 rpm) for 27 s. After 21 s from the second spinning step, 150 μL of chlorobenzene was dropped onto the sample as an antisolvent. Prior to the 30 min annealing at 100 °C, the samples looked semitransparent with a dark brown tone. At the end of the annealing process, the films were black with a shiny, mirror-like appearance. For the ETL, 27 nm of C_60_, 8 nm of BCP, and 100 nm of Ag were sequentially thermally evaporated onto the films at a base pressure of ∼10^–6^ mbar at a rate of 0.1 nm s^–1^. The masks used to deposit the Ag layer resulted in six active pixels on each substrate, each with an active area of 0.045 cm^2^. All film deposition steps were carried out in a N_2_-filled glovebox.

### Current Density–Voltage Measurements

The current density–voltage (*J*–*V*) measurements were obtained using a Keithley 4200 Source-Measure unit with a scan rate of 25 mV s^–1^. For light measurements, samples were irradiated with an Oriel Instruments Solar Simulator (xenon lamp) to simulate AM1.5G irradiance. The lamp was calibrated with a silicon reference cell. A Thorlabs green light, 530 nm LED, powered by a function generator (Thorlabs, DC22000), and optical density filters (Thorlabs) of 1.0, 2.0, 3.0, and 4.0 were used to carry out the linear dynamic range (LDR) measurements.

### Noise Measurements

The spectral density of the device noise was measured using a digital oscilloscope (Siglent, SDS6054A) in dark conditions with the aid of a high-speed current amplifier (Femto, DHPCA-100). A fast Fourier transform was then carried out to obtain the noise spectrum.

Cutoff frequency was measured using a digital oscilloscope (Siglent, SDS6054A), which had a 530 nm Thorlabs diode connected to a Siglent SDG1000X Series Function generator. The frequency range spanned from 100 Hz to 100 kHz.

For determination of the rise and fall time, a 5 kHz square wave pulse was applied to the LED using the function generator.

### EQE and Responsivity

EQE and responsivity were measured using a Quantum Design, PV300, after the light signal was calibrated with a reference silicon photodiode (Thorlabs, S120VC).

### Spectroscopy Measurements

A Cary 60 UV–vis Agilent spectrophotometer was used to record the absorption spectra.

### Air Photoemission Spectroscopy

Measurements were carried out with a KP Technology, SKP5050/APS02. SAMs were drop-cast on ITO substrates.

### Morphological Analysis

Scanning electron microscopy (SEM) images were acquired by using a Zeiss Gemini 1 Sigma 300 field emission scanning electron microscope. The operation voltage varied between 1 and 5 kV.

X-ray diffraction (XRD) was carried out to determine the active layer’s crystal structure and the various phases present within it. A Bruker D2 Phaser was equipped with a Cu X-ray source (λ = 1.54060 Å).

Contact angle measurements were recorded using an Oscilla contact angle goniometer, with a 4:1 mixture of DMF:DMSO being dropped on the SAM covered substrates.

An Agilent 5100 AFM/SPM microscope was used to take the AFM images with a 5 μm × 5 μm range. The film’s rough and hard nature meant that an oscillation amplitude of 7.0 V was used, and a slow scan speed of 0.7 line/s was used to take the measurements. The images were plotted and analyzed using Gwyddion.

### Impedance Spectroscopy

A Metrohm μStat-i 400 BiPotentiostat/Galvanostat/Impedance analyzer was used to measure impedance over a 1 MHz to 1 Hz range. The devices were illuminated with a 530 nm Thorlabs LED (driven by a Thorlabs DC22000 function generator) while subjected to a 1.0 V DC bias and a superimposed 20 mV AC voltage. The resistance and chemical capacitance values were then extracted using the “EIS Spectrum Analyser” software. All values had a ≤5% error associated.

### Time-Resolved Photoluminescence

TRPL measurements were conducted using a Horiba Delta Flex system (detector: PPD-900, Horiba Scientific). A 467 nm laser diode with <200 ps pulse duration (NanoLED N-02B, Horiba Scientific) was used to achieve the excitation with a repetition rate of 1 MHz and a fluence of 0.64 nJ cm^–2^ pulse^–1^.

## Supporting Information Available

Air photoemission spectroscopy; absorption spectroscopy; work function measurements; SEM; AFM; XRD; *J*_d_ statistics; LDR, cutoff frequency, and rise/fall time measurements; *D** plots; TCSPC data; EIS (DOCX)
