# Supplementary material for: Blending Self-Assembled Monolayers for Enhanced Band Alignment and Improved Morphology in p-i-n Perovskite Photodetectors
Source: ACS Appl Mater Interfaces. 2024 Jun 19;16(26):33838–45. doi: 10.1021/acsami.4c06447 (PMC11231979; doi:10.1021/acsami.4c06447)
Supplement: Supplementary file 1 — am4c06447_si_001.docx [file am4c06447_si_001.docx]

**Supporting information**

**Blending self-assembled monolayers for enhanced band alignment and improved morphology in p-i-n perovskite photodetectors**

Edoardo Angela^1^, Davide Nodari^2^, Francesco Furlan^2^, Julianna Panidi^2^, Martyn A. McLachlan^1^* and Nicola Gasparini^2^*

^1^Department of Materials, Molecular Science Research Hub, Imperial College, London, W12 0BZ, UK

^2^Department of Chemistry and Centre for Processable Electronics, Molecular Science Research Hub, Imperial College, London, W12 0BZ, UK

[martyn.mclachlan@imperial.ac.uk](mailto:martyn.mclachlan@imperial.ac.uk)

[n.gasparini@imperial.ac.uk](mailto:n.gasparini@imperial.ac.uk)

Figure S1: APS data for drop casted SAM solutions on ITO, the x-axis intersect with the abscissa indicates the HOMO energy.

Figure S2: APS data for the CsFAMA perovskite active layer, the x-axis intersect with the abscissa indicates the VB energy.

Figure S3: a) UV-Vis plot and b) Tauc plot used to measure bandgap energy for the CsFAMA perovskite active layer

Table S1: Table reviewing the various HOMO levels and work functions for each condition. ITO substrates were spin coated with the various HTLs to extract work functions, whereas drop casting was used to extract HOMO levels. Bare ITO has also been reported.

| **Sample** | **HOMO (eV)** | **Work Function (eV)** |
| --- | --- | --- |
| Me-4PACz | -5.7 | 5.32 |
| Blend | -5.8 | 5.29 |
| 2-PACz | -6.0 | 5.29 |
| ITO | - | 4.77 |

Figure S4: Top view SEM images of the various perovskite films grown on ITO + SAM. While no visible change in grain size is seen, defects such as pinholes can be observed in for films grown on Me-4PACz

Figure S5: AFM plots for the various conditions on the same 250 nm scale. Note the Me-4PACz has a roughness more than double that for the other two conditions

Figure S6: XRD plots for the triple cation perovskite grown on ITO + SAM for the various conditions. A δ-FAPI peak is observed at 11.7° due to the incomplete conversion of the FA based perovskite to the photoactive layer REF

Figure S7: Statistical J_d_ distributions for the SAM compositions used. The J_d_ values for other SAM blend ratios used during the optimization have been included.

Figure S8: LDR plot for the different conditions. The calculated slope for each linear region in the plot was of 1.00 ± 0.02

Figure S9: D* plot using J_d_ for i_n_ calculation rather than measuring i_n_, the result is a two-fold overestimation of the true value.

Figure S10: Equivalent circuit used to fit Nyquist plots

Figure S11: TCSPC data obtained for the triple cation films grown on ITO + SAM.

Figure S12: Cut-off plot for the various SAM conditions. Me-4PACz, Blend and 2-PACz conditions revealed cut-off values of 224 kHz, 398 kHz and 355 kHz respectively.

Figure S13: Rise and Fall plot revealing rise times of 10.4 μs, 2.1 μs and 1.9 μs for Me-4PACz, Blend and 2-PACz conditions. Fall times were found to be 11.4 μs, 2.2 μs and 1.8 μs respectively.

Table S2: Table reporting the values fitted using the EIS software. All values were within a 5% error.

| **Sample** | **C_μ_ (F)** | **R_ext_ (Ω)** | **R_rec_ (Ω)** | **τ_ext_ (μs)** | **τ_rec_ (μs)** |
| --- | --- | --- | --- | --- | --- |
| Me-4PACz | 7 × 10^-9^ | 97 | 80 | 0.68 | 0.56 |
| Blend | 6 × 10^-9^ | 30 | 94 | 0.27 | 0.85 |
| 2-PACz | 9 × 10^-9^ | 46 | 105 | 0.28 | 0.63 |

Table S3: lifetime of the overall decay processes τ_avg_ for the various conditions. The τ_avg_ values were calculated using the equation $\tau_{avg}= \frac{A_{1}\tau_{1}^{2}+ A_{2}\tau_{2}^{2}}{A_{1}\tau_{1}+ A_{2}\tau_{2}}$ according to the methodology used in ^53^, where a double exponential decay function is fit to the decay curve to account for the decay mechanisms.

| **Sample** | **A_1_** | **τ_1_** | **A_2_** | **τ_2_** | **τ_avg_ (ns)** |
| --- | --- | --- | --- | --- | --- |
| Me-4PACz | 0.10 | 0.52 | 0.011 | 139 | 134 |
| Blend | 0.14 | 0.56 | 0.032 | 168 | 164 |
| 2-PACz | 0.11 | 0.54 | 0.012 | 153 | 147 |
